# Emotional well‐being and work engagement of nurses who moonlight (dual employment) in private hospitals

**DOI:** 10.1111/ijn.12783

**Published:** 2019-09-12

**Authors:** Michelle Engelbrecht, Asta Rau, Petrus Nel, Marisa Wilke

**Affiliations:** ^1^ Centre for Health Systems Research & Development University of the Free State Bloemfontein South Africa; ^2^ Department of Industrial Psychology University of the Free State Bloemfontein South Africa; ^3^ School of Nursing University of the Free State Bloemfontein South Africa

**Keywords:** nurses, well‐being, work engagement

## Abstract

**Background and aim:**

Given the myriad occupational stressors of nursing itself, plus the challenges of moonlighting, we aimed to investigate the emotional well‐being of moonlighting nurses and their work engagement. Well‐being was defined by levels of general health, mental health, emotional exhaustion, personal accomplishment, compassion satisfaction and compassion fatigue.

**Design:**

A cross‐sectional descriptive survey (December 2017 to March 2018) at private health care facilities in a Metropolitan Municipality, South Africa.

**Methods:**

Two hundred and fifty‐one nurses completed self‐administered questionnaires, which comprised of validated scales.

**Results:**

Nurses were at low risk for emotional exhaustion (M=12.8; SD=11.23) and scored high on compassion satisfaction (M=42.34; SD=7.22) and work engagement (M=4.87, SD=1.18). Personal accomplishment (*t*= 2.535; *P*<.05) compassion satisfaction (*t*= 6.790; *P*=.000) and mental health (*t*=3.206; *P*<.05) made a statistically significant unique contribution to the prediction of work engagement. Nurses who had considered leaving the profession scored significantly higher on emotional exhaustion and compassion fatigue.

**Conclusion:**

Nurses who moonlighted in private health care facilities reported low risk for burnout and high levels of compassion satisfaction and work engagement. Further research is needed to explore the reasons for these findings. Attention must be given to ensuring the occupational well‐being of nurses in order to retain them in the profession.

## SUMMARY STATEMENT

What is already known about this topic?

- Nursing is a stressful occupation.
- Moonlighting (dual employment) is fraught with challenges for nurses as individuals and for institutions where they work.
- Very little is known about the emotional well‐being of nurses who moonlight.

What this paper adds?

- This is the first cross‐sectional study on emotional well‐being and work engagement of nurses who moonlight in private hospitals in South Africa.
- Nurses moonlighting in private health care facilities reported low risk for burnout and high levels of compassion satisfaction and work engagement
- Moonlighting nurses who had considered leaving nursing scored higher on emotional exhaustion and compassion fatigue than those who had *not* considered leaving nursing.

The implications of this paper:

- Better‐resourced private health care facilities may contribute towards the finding that nurses moonlighting in private health care facilities reported low risk for burnout and high levels of compassion satisfaction.
- Attention must be given to ensure the occupational well‐being of nurses in order to retain them in the profession.
- The finding that lower categories of nurses score higher on work engagement than professional nurses requires further research.

## 1 INTRODUCTION

In South Africa, as elsewhere, nurses constitute the single largest group of health care workers (HCWs) (76.8%), and the importance of their role in promoting health and providing services is undisputed (Department of Health, 2011; Khunou, & Davhana‐ Maselesele, M., 2016; Mayosi & Benatar, 2014; Rispel & Bruce, 2015). According to Van Rensburg (2014) and Rispel (2016), while South Africa has higher ratios of health professionals than the World Health Organization's minimum norms, the country is confronted with a nursing crisis. This crisis is “… characterised by shortages, declining interest in the profession, lack of a caring ethos, and an apparent disjuncture between the needs of nurses on the one hand and those of communities served on the other hand” (Rispel & Bruce, 2015: 118). These shortages include maldistribution of HCWs between urban and rural areas, as well as between public and private health care facilities (Rispel, 2016; Van Rensburg, 2014), inappropriate skills mixes and an exodus of health professionals to developed countries. This renders unequal allocation of health care along private‐public, urban‐rural, wealthy‐poor, medically insured‐state dependent lines (Van Rensburg, 2014).

The South African government devised numerous strategies to combat nurse shortages including rural and scarce‐skills allowances to address the dual private‐public and rural‐urban inequity, occupation‐specific dispensation to improve service conditions and remuneration, increased intake of nursing students (Van Rensburg, 2014), and allowing nurses to enter dual practices (Muller & Seekoe, 2014; Van Rensburg, 2014). Flexible work arrangements have emerged as an important strategy to address nursing shortages. This “casualization” of the nursing workforce entails employing nurses on short‐term contracts without benefits. There are different types of casual work arrangements: one is moonlighting, where nurses have a full‐time and one or more temporary paid nursing job(s) (Rispel & Blaauw, 2015).

Public servants in South Africa are permitted to engage in “Other remunerative work” (RWOPS); however, provincial departments of health report that RWOPS is widely abused and requires careful management (Department of Health, 2011). A cross‐sectional survey across four provinces of South Africa found that moonlighting and agency nursing were widespread, with 28% of nurses moonlighting in the 12 months preceding the survey, mainly for financial gain. Furthermore, the prevalence of moonlighting among nurses working in private health facilities (40.6%) was higher in comparison to nurses in public health care (24.2%) (Rispel, Blaauw, Chirwa, & de Wet, 2014).

Moonlighting is fraught with challenges for nurses as individuals and for institutions where they work (Muller & Seekoe, 2014; Rispel et al., 2014; Van Rensburg, 2014). Local research found that nurses were more likely to take sick leave when not sick in order to moonlight at another facility and paid less attention to nursing work while on duty (Rispel & Blauuw, 2015). Challenges in interpersonal relationships between permanently employed nurses and moonlighting nurses also surfaced (Muller & Seekoe, 2014): moonlighters were seen as unreliable, with poor attitudes—including reluctance to take on extra duties, and judged as giving poor quality of care (Rispel & Moorman, 2015).

Given the myriad occupational stressors of nursing itself, from personal through to systemic (Adzakpah, Laar, & Fiadjoe, 2016; Coetzee, Klopper, Ellis, & Aiken, 2013; Khamisa, Peltzer, Ilic, & Oldenburg, 2017; Khamisa, Peltzer, & Oldenburg, 2013; Khunou, & Davhana‐ Maselesele, M., 2016; Van der Heijden, Brown, & Xu, 2019; Wentzel & Brysiewicz, 2018) plus the challenges of moonlighting, we question the emotional well‐being of moonlighting nurses and their work engagement. Although there are studies on the emotional well‐being of nurses in general (Coetzee et al., 2013; Gómez‐Urquiza et al., 2019; Khamisa, Oldenburg, Peltzer, & Ilic, 2015; Lizano, 2015; Monsalve‐Reyes et al., 2018; Pradas‐Hernández et al., 2018; Van der Colff & Rothmann, 2014), very little is known about the many who moonlight (Rispel et al., 2014; Russo, Fronteira, Jesus, & Buchan, 2018).

This paper investigates some of the debilitating effects of nursing on work engagement. In this regard, we looked at levels of burnout and compassion fatigue, two distinct yet related occupational stress syndromes. Burnout is a combination of negative behavioural, attitudinal and physical changes in response to work‐related stress (Schaufeli & Bakker, 2004) and comprises of emotional exhaustion (burnout in the first stage) followed by depersonalization (used as a coping strategy). Thereafter, feelings of reduced personal accomplishment may be experienced. Compassion fatigue is primarily associated with the potentially adverse emotional effects of empathetic engagement with patients and from direct exposure to disturbing events (Stamm, 2005).

Both burnout and compassion fatigue are associated with negative outcomes for individuals, organizations and the quality of service provision. It is proposed that low levels of burnout and compassion fatigue and high levels of compassion satisfaction and personal accomplishment will result in higher levels of work engagement. Work engagement is a fulfilling, positive state of mind characterized by three dimensions: (a) vigour (high levels of energy and mental resilience at work), (b) dedication (finding significance in one's work), and (c) absorption (total and happy immersion in one's work). Nurses' work engagement is closely associated with predictions of burnout (Schaufeli & Bakker, 2004), well‐being (Kanste, 2011) and staff retention (Brunetto et al., 2013).

## 2 METHODS

### 2.1 Ethical considerations

Ethical clearance was obtained from an Ethics Committee based at a university within the study setting. The various scales were purchased/accessed in line with their copyright agreements.

### 2.2 Aim and objectives

The study aimed to investigate emotional well‐being and work engagement of nurses who moonlight in private hospitals in a Metropolitan Municipality in South Africa. Well‐being was defined by levels of general health, mental health, emotional exhaustion, personal accomplishment, compassion satisfaction and compassion fatigue. The objectives of the study were the following:

- Describe levels of general health, emotional exhaustion, personal accomplishment, compassion fatigue, compassion satisfaction and work engagement experienced by moonlighting nurses.
- Compare levels of emotional exhaustion, compassion fatigue and work engagement of moonlighting nurses who had considered and who had *not* considered leaving the nursing profession.
- Compare work engagement scores of professional, enrolled and assistant nurses.
- Determine the influence of personal accomplishment, compassion satisfaction, role functioning and mental health on work engagement of moonlighting nurses.

### 2.3 Design

A cross‐sectional descriptive survey was undertaken at private health care facilities in a Metropolitan Municipality, South Africa. South Africa has a two‐tiered health care system comprising of an under‐resourced public sector and a well‐resourced private sector. The majority of South Africans (84%) access health care through government‐run facilities, which are underfunded and understaffed. The public health system is tax funded—primary health care services are offered for free, while free hospital services are subject to a means test. Anyone can access public and private health services; however, access to private health care depends on the individual's ability to pay for these services. Therefore, the wealthiest 20% of the population uses the private system and is far better served (Benatar, Sullivan, & Brown, 2017).

### 2.4 Sample

Hospital management at the 12 main private health care facilities in the Metro were approached and permission sought for the study. Only one facility refused to participate. Purposive sampling was used to identify nurses who moonlight at these facilities. Three hundred and fifty‐five questionnaires were distributed, of which 282 (79% response rate) were returned. Thirty‐one questionnaires were discarded due to extensive missing data, leaving a total of 251 completed questionnaires. In our study, 49% of the respondents were professional nurses (four years of training), 27.9% were assistant nurses (one year of training) and 23.1% were enrolled nurses (two years of training). The majority were female (82.5%), and the average age was 40.51 years (SD 11.72).

### 2.5 Data collection

Due to the sensitive nature of moonlighting (some nurses prefer not to disclose that they moonlight), the nursing services managers at each of the facilities distributed the questionnaires. Moonlighting nurses were given an envelope containing the informed consent form and questionnaire, which they completed on their own time. The sealed envelope was returned and placed in a box in the nursing services manager's office. The researchers collected the sealed envelopes on a weekly basis. Fieldwork took place over a four‐month period (December 2017 to March 2018).

### 2.6 Measures

The first section of the questionnaire collected demographic and background information (eg, sex, age, marital status, dependents, occupational category, place of employment, work experience reasons for moonlighting and intention to leave nursing). The second section comprised of standardized and validated scales from the following instruments: Maslach Burnout Inventory ‐ Human Services Survey (Maslach, Leiter, & Jackson, 1996), Professional Quality of Life Scale (Stamm, 2005), Medical Outcomes Study (MOS) 20‐Item Short‐Form Health Survey (Rand Corporation, 2017) and the Utrecht Work Engagement Scale (Schaufeli & Bakker, 2004). The respondents were asked to confine responses to their experiences in the hospitals where they moonlighted.

The Maslach Burnout Inventory comprises 22 items designed to assess three dimensions of burnout: emotional exhaustion, depersonalization and personal accomplishment. High scores on emotional exhaustion and depersonalization and low scores on personal accomplishment are associated with high levels of burnout. The items are scored on a 7‐point Likert scale ranging from zero (never) to 6 (every day), indicating the frequency of feelings and attitudes experienced. Cronbach alpha coefficients ranging from.71 to.90 were reported (Maslach et al., 1996). Reliability and validity were also found acceptable in South African studies (Coetzee et al., 2013; Van der Colff & Rothmann, 2014). Scoring is as follows: emotional exhaustion – low score ≤18, average score 19 to 26, high score ≥27; depersonalization – low score ≤5, average score 6 to 9, high score ≥10; and personal accomplishment – low score ≥40, average score 39 to 40, high score ≤33 (Maslach et al., 1996).

The Professional Quality of Life Scale (ProQOL) comprises 30 items for measuring compassion fatigue, burnout and compassion satisfaction. Respondents reflect on how frequently they experienced situations in their work setting over the last 30 days, using a 5‐point Likert scale ranging from 1 (never) to 5 (very often). Stamm (2005) reports alpha coefficients higher than.74 for all three subscales. South African studies with nurses report good levels of internal consistency for two subscales: compassion satisfaction and compassion fatigue (Mashego, Nesengani, Ntuli, & Wyatt, 2016; Wentzel & Brysiewicz, 2018). For all scales, a low‐risk cut‐off score was set at total sum of 22 or less, between 23 and 41 for average risk, and 42 or more for high risk (Stamm, 2005).

The MOS 20‐Item Short‐Form Health Survey (SF‐20) is a short multidimensional instrument, which measures six aspects of health status: physical functioning, role functioning, social functioning, mental health, general health perceptions and bodily pain (Rand Corporation, 2017). The internal consistency of the subscales was established in earlier studies among elderly and patient populations (Carver, Chapman, Thomas, Stadnyk, & Rockwood, 1999; Holmes, Bix, & Shea, 1996) but has not been tested for reliability amongst South African nurses. Scores are reversed so that a high value indicates better functioning (Rand Corporation, 2017).

The short version of the Utrecht Work Engagement Scale comprises of nine items and three subscales: vigor, dedication and absorption. Respondents reflect on how often they feel a certain way about their work using a 7‐point Likert scale ranging from 0 (never) to 6 (every day). It is reported that work engagement may be considered a one‐dimensional as well as a three‐dimensional construct (Schaufeli & Bakker, 2004). A South African study among working adults found high correlations between the three dimensions and the high values for Cronbach's alpha for the total scale (0.92), supporting a one‐dimensional model (De Bruin & Henn, 2013). Higher scores indicate higher levels of work engagement (Schaufeli & Bakker, 2004).

### 2.7 Data analysis

Data were double captured, cleaned and analysed in IBM SPSS version 25. Descriptive statistics were generated yielding frequency counts and percentages for categorical variables, and means and standard deviations for continuous variables. Composite scores were calculated for all subscales. Cronbach's alpha was used to test the internal consistency of the scales and subscales. The independent sample *t* test was used to determine if there was a difference between (a) the mean emotional exhaustion scores between respondents who had/had not considered leaving nursing, (b) the mean compassion fatigue scores between respondents who had/had not considered leaving nursing, and (c) the mean work engagement scores between respondents who had/had not considered leaving nursing. One‐way analysis of variance was used to determine if there was a difference between the mean work engagement scores of professional, enrolled and assistant nurses. Standard multiple regression was performed to predict work engagement from personal accomplishment, compassion satisfaction, role functioning and mental health.

## 3 RESULTS

### 3.1 Biographic and employment characteristics

Slightly more than half of the respondents were married (n=130; 53.5%); with most reporting biological dependants (n=203; 81.5%). On average, respondents were responsible for 2.03 (SD 1.143) biological dependents and 2.46 “other” dependants (SD 2.390). The majority of respondents indicated that their current primary position was in a private health care facility (n=184; 83.6%); 11% (n=24) did not work full‐time in a hospital, and 5.4% (n=12) worked in public hospitals. In their primary employment positions, 44.6% of respondents worked during the day (n=111), 18.9% worked at night (n=47) and 36.5% worked both day and night shifts (n=91).

All respondents moonlighted during 2017. On average, respondents moonlighted for 5.53 years (SD 5.553), for financial reasons (n=177; 79.0%), the opportunity to learn new skills (n=150; 77.3%) and job variety (n=147; 78.6%). A fifth of the respondents (n=55; 22.3%) had considered leaving nursing during the past year. The most common reasons for considering leaving nursing included financial (n=28; 24.8%), workload (n=18; 15.9%), nurses are not valued (n=13; 11.5%), stress (n=9; 8.0%) and the desire to study further (n=8; 7.08%).

### 3.2 Reliability of the scales

An alpha value of.7 is considered a sufficient measure of reliability (Taber, 2018). For these reasons, scales with an alpha <.7 were eliminated from further analyses (see Table 1): depersonalization scale (Maslach Burnout Inventory), burnout scale (Professional Quality of Life) and health perceptions (MOS Short Form).

**Table 1:** Reliability of the scales

Scales	No of Items	Cronbach's Alpha
Maslach Burnout Inventory	22	
‐ Emotional Exhaustion (EE)	9	.9
‐ Depersonalization (DP)	5	.4
‐ Personal accomplishment (PA)	8	.8
Professional Quality of Life	30	
‐ Compassion satisfaction (CS)	10	.9
‐ Burnout (B)	10	.5
‐ Compassion fatigue (CF)	10	.8
MOS Short Form	20	
‐ Physical functioning	6	.9
‐ Role functioning^a^	2	‐
‐ Social functioning^a^	1	‐
‐ Mental health	5	.8
‐ Health perceptions	5	.5
‐ Pain^a^	1	‐
Utrecht Work Engagement Scale	9	.9
‐ Vigour	3	.7
‐ Dedication	3	.8
‐ Absorption	3	.7

a Cronbach's alpha was not calculated for scales with two items and less.

### 3.3 General health

A third of the respondents (n=93; 37.1%) reported that they were in poor health or that their health was “fair” (n=92; 36.6%), while 26.3% reported good to excellent health (n=66). Despite a third reporting poor health, the nurses scored high on physical functioning, with an average of 10.84 (SD=1.92; range 6‐12). Some problematic areas in terms of health limitations, reported by a fifth of the respondents, were vigorous activities such as lifting heavy objects, running or participating in strenuous sports (n=75; 29.9%); walking up hill or climbing stairs (n= 59; 23.5%); and bending, lifting or stooping (n=52; 20.7%). The nurses scored an average of 23.6 (SD=4.78, range 4‐24) on the mental health scale, indicating good mental health. A closer examination of the individual items revealed that a fifth of the respondents sometimes felt nervous (n=53; 21.1%) and/or depressed (n=61; 24.3%) (see Table 2).

**Table 2:** Levels of general health, burnout and compassion fatigue

Scales	Range	Mean	SD
MOS Short Form			
‐ Physical functioning	6‐12	10.84	1.92
‐ Mental health	4‐24	23.60	4.78
Maslach Burnout Inventory			
‐ Emotional exhaustion	0‐45	12.80	11.23
‐ Personal accomplishment	0‐40	34.99	10.82
Professional Quality of Life			
‐ Compassion satisfaction	10‐50	42.34	7.22
‐ Compassion fatigue	10‐50	21.18	6.42
Utrecht Work Engagement Scale		4.87	1.18
‐ Vigour	0‐6	4.64	1.39
‐ Dedication	0‐6	5.33	1.19
‐ Absorption	0‐6	4.63	1.45

### 3.4 Burnout

The nurses scored an average of 12.8 (SD=11.23, range 0‐54) on the Emotional Exhaustion subscale, indicating low risk for burnout. A categorical breakdown of the subscale revealed that 70.9% (n=178) scored low on emotional exhaustion, while 13.1% (n=33) had a high score. There was a statistical significant difference in the mean emotional exhaustion scores between respondents who considered and those who had *not* considered leaving nursing. Respondents who considered leaving nursing scored higher on emotional exhaustion (M=19.10, SD=12.20) than those who had not (M=10.97; SD=10.31), 95% confidence interval [4.89, 11.38], *t*(245) = 4.943, *P*=.000.

At the other end of the spectrum, the nurses scored an average of 34.99 (SD=10.82, range 0‐48) on personal accomplishment, indicating an overall average score. More specifically, 34.3% of respondents (n=86) scored high on personal accomplishment compared with 43.8% (n=110) who scored low on this sub‐scale (see Tables 2 and 3).

**Table 3:** Categorization of general health, burnout and compassion fatigue

Scales	Low		Average		High	
	N	%	N	%	N	%
Maslach Burnout Inventory						
‐ Emotional exhaustion	178	70.9	40	15.9	33	13.1
‐ Personal accomplishment	110	43.8	55	21.9	86	34.3
Professional Quality of Life						
‐ Compassion satisfaction	6	2.4	80	31.9	165	65.7
‐ Compassion fatigue	153	61.0	96	38.2	2	0.8
Utrecht Work Engagement Scale	15	6.0	6	2.4	230	91.6

### 3.5 Compassion fatigue and satisfaction

An average of 21.8 (SD=6.42) was scored on the Compassion Fatigue subscale, an indication of low levels of compassion fatigue. More specifically, a categorical breakdown of the scale revealed that 61% had a low score on compassion fatigue and 38.2% had an average score. There was a statistical significant difference in the mean compassion fatigue scores between respondents who considered and who had *not* considered leaving nursing. Respondents who considered leaving nursing scored higher on compassion fatigue (M=23.34, SD=7.14) than those who had not (M=20.54; SD=6.09), 95% confidence interval [0.891,4.711], t(245) = 2.889, p=.004.

At the opposite end of the spectrum, respondents scored an average of 42.34 (SD=7.22) on the Compassion Satisfaction subscale indicating high levels of compassion satisfaction. A categorical breakdown of these scores revealed that 65.7% of the nurses (n=165) had a high score on compassion satisfaction and 31.9% (n=80) had an average score (see Table s2 and 3).

### 3.6 Work engagement

The respondents scored high on work engagement (M 4.87, SD=1.18) (see Table 2). There was a statistical significant difference in the mean work engagement scores between respondents who considered and who had *not* considered leaving nursing. Respondents who considered leaving nursing scored lower on work engagement (M=4.31, SD=1.19) than those who had not (M=5.05; SD=1.08), 95% confidence interval [‐1.088, ‐0.291], *t*(245) = ‐3.449, *P*=.001.

There was a statistically significant difference in work engagement scores between different categories of nurses, *F*(2,248) = 4.168, *P*<.05. Tukey post‐hoc analyses revealed that there was a statistically significant difference in work engagement between professional nurses and assistant nurses (*P*=.017). Assistant nurses scored higher on work engagement (M=5.14, SD=1.097) than professional nurses (M=4.66, SD=1.24). No other group differences were statistically significant.

Multiple regression was run to predict work engagement from personal accomplishment, compassion satisfaction, role functioning and mental health (see Table 4). The assumptions of linearity, independence of errors, homoscedascity, unusual points and normality of residuals were met. These variables statistically significantly predicted work engagement (*F*(4, 246) = 32.546, *P*=.000, adjusted *R* ^2^ = 0.335). Personal accomplishment (*t*= 2.535; *P* <.05) compassion satisfaction (*t*= 6.790; *P*=.000) and mental health (*t*=3.206; *P*<.05) made a statistically significant unique contribution to the prediction of work engagement. Compassion satisfaction (β = .420) was the highest predictor of work engagement followed by mental health (β = .180) and finally personal accomplishment (β = .146).

**Table 4:** Standard multiple regression analysis related to the prediction of work engagement

Independent variables	*B*	Std Error	*β*	*t*	*P*
Personal accomplishment	.016	.006	.146	2.535	.012
Compassion satisfaction	.069	.010	.420	6.790	.001
Mental health	.045	.014	.180	3.206	.002
Physical functioning	‐.002	.033	‐.004	‐.067	.946

## 4 DISCUSSION

This is the first cross‐sectional study on emotional well‐being and work engagement of nurses who moonlight in private hospitals in South Africa. Although there is an abundance of research on emotional well‐being of nurses (Van der Colff & Rothmann, 2014; Khamisa et al., 2015; Khunou, & Davhana‐ Maselesele, M., 2016; Makhado & Davhana‐Maselesele, 2016; Mashego et al., 2016; Dlamini & Visser, 2017; Roomaney, Steenkamp, & Kagee, 2017; Wentzel & Brysiewicz, 2018) in South Africa, there are no studies specifically focusing on emotional well‐being of nurses who moonlight. We defined well‐being by investigating levels of general health, mental health, emotional exhaustion, personal accomplishment, compassion satisfaction and compassion fatigue.

The overall psychosocial well‐being of the respondents was good, and they had high levels work engagement. More specifically, our respondents were at low risk for emotional exhaustion, which is the first stage of burnout. This is in contrast to findings of other South African studies, which reported higher levels of emotional exhaustion among nurses (Makhado & Davhana‐Maselesele, 2016; Van der Colff & Rothmann, 2014). More specifically high levels of emotional exhaustion were found: (a) among 34.6% of registered nurses in the private, public, hospital, community, psychiatric and management sectors of seven provinces of South Africa (Van der Colff & Rothmann, 2014) and (b) among 53% of nurses caring for people living with HIV/AIDS at a regional hospital (Makhado & Davhana‐Maselesele, 2016). Given the low scores on emotional exhaustion (ie, low risk for burnout), we were not surprised to see that our respondents had average scores on feelings of personal accomplishment, a further indication of low risk for burnout.

These findings suggest that the respondents, in general, were not experiencing burnout, which was also supported by low scores on compassion fatigue and high levels of compassion satisfaction. Again, this is in contrast to findings from other South African studies among nurses (a) with exposure to maternal and perinatal deaths and (b) working in oncology, who reported average to high levels of compassion fatigue (Mashego et al., 2016; Wentzel and Brysiewicz (2018). Compassion fatigue is related to potentially adverse emotional effects that may result from empathetic engagement with patients and from direct exposure to disturbing events (Stamm, 2005). The higher levels of compassion fatigue reported by Mashego et al. (2016) could be due to the nature of the work (ie, maternal and perinatal deaths) that the nurses engaged in. On the other hand, the high levels of compassion satisfaction scored by our nurses indicate that they derived pleasure from doing their work and considered themselves to be effective caregivers (Stamm, 2005). Similar findings were reported by Moshego et al. (2016) and Wentzel and Brysiewicz (2018), which they interpret as possibly being attributed to personal resilience and good social support networks coupled with self‐care.

Although the respondents appeared to have high levels of well‐being, those nurses who had considered leaving nursing in the year prior to the study scored higher on emotional exhaustion and compassion fatigue than those who had *not* considered leaving nursing. This is a clear indication of the importance of taking care of the well‐being of nurses in order to retain this scarce human resource, particularly as nursing in South Africa is in a crisis (Rispel & Bruce, 2015).

Overall, our findings were positive despite numerous occupational stressors that nurses are confronted with (Coetzee et al., 2013; Khamisa et al., 2013; Khamisa et al., 2017; Khunou, & Davhana‐ Maselesele, M., 2016; Wentzel & Brysiewicz, 2018) plus the challenges of moonlighting (Muller & Seekoe, 2014; Rispel & Blaauw, 2015). A possible explanation is that our respondents moonlighted in private hospitals. Previous research found that nurses preferred to moonlight in private hospitals as they are clean and well resourced (Muller & Seekoe, 2014). Coetzee et al. (2013) concur that more favourable work environments are significantly associated with more positive nurse reported quality of care, and nurse workforce outcomes. Furthermore, they reported that high burnout in nurses was significantly more common in public than private hospitals. Similar results were reported in a study comparing work satisfaction of professional nurses working in private and public hospitals across South Africa. Nurses in private hospitals were found to be more satisfied with their work than their counterparts in the public sector (Pillay, 2009).

With regard to work engagement, we proposed that low levels of burnout and compassion fatigue and high levels of compassion satisfaction and personal accomplishment would result in higher levels of work engagement. This hypothesis was confirmed by our findings. Overall the respondents scored high on work engagement, which is assumed to be the opposite of burnout. More specifically, we found that high levels of personal accomplishment, compassion satisfaction and mental health made a statistically significant unique contribution to the prediction of work engagement. Similar findings were reported by Mason et al. (2014), who found significant positive correlations between compassion satisfaction and work engagement. Assistant nurses scored higher on work engagement than professional nurses. Similar results were reported from a study among nurses in a Cairo hospital, where staff nurses scored higher on work engagement than head nurses (Seada, 2017) and from a South African study where auxiliary/assistant nurses also scored higher on work engagement than professional nurses (D'Emiljo & Du Preez, 2017). We also found that respondents who considered leaving nursing scored lower on work engagement than those who had *not* considered leaving the profession.

### 4.1 Limitations

The study was conducted in one metropolitan municipality among a purposive sample of nurses who moonlight in private hospitals; therefore, the results cannot be generalized to other settings. As with most self‐reported measures, some level of response bias is likely; it could be helpful to supplement survey data with other data sources. Nurses were requested to confine their answers to the facility where they moonlighted. However, we realize that this may not always have been the case.

## 5 CONCLUSION

In general, nurses who moonlighted in private health care facilities reported low risk for burnout, and high levels of compassion satisfaction and work engagement. It is recommended that further qualitative research is necessary to explore reasons why nurses who moonlight in private health care facilities experience the levels of emotional well‐being that they do. Furthermore, nurses who had considered leaving the nursing profession in the last year had higher levels of burnout and compassion fatigue and illustrate how important it is for health care facilities and managers to focus on staff well‐being in the workplace. It is also recommended that further research is needed to explore why lower categories of nurses scored higher on work engagement.

## FUNDING

Funding from the University of the Free State (Interdisciplinary Grant) is gratefully acknowledged.

## CONFLICT OF INTEREST

The authors declare no conflict of interest.

## AUTHORSHIP STATEMENT

ME, AR, PN and MW designed the study and research instruments. ME analysed the data and prepared the manuscript. AR, MW and PN gave inputs on the manuscript. All authors approved the final version for submission.

## AVAILABILITY OF MATERIAL

Data from the current study are available from the corresponding author on reasonable request.
